# Comparison of the Efficacy of Two Local Haemostatic Agents

**DOI:** 10.5812/kowsar.22517464.2850

**Published:** 2012-01-15

**Authors:** Nader Nowshad, Masoud Saghafinia, Farzad Panahi, Shahram Bolandparvaz, Nader Tanideh

**Affiliations:** 1 Trauma Research Center ,Shiraz Medical School , Shiraz University of Medical Sciences, Shiraz, IR Iran; 2 Trauma Research Center, Baqiyatallah University of Medical Sciences, Tehran, IR Iran; 3 Trauma Research Center, Shiraz University of Medical Sciences, Shiraz, IR Iran; 4 Shiraz University of Medical Sciences, Shiraz, IR Iran

**Keywords:** Trauma, Models, Animal

## Abstract

**Background:**

In modern life, the incidence of traumatic injuries increases daily. In accidents which lead to trauma, massive bleeding is the main cause of death. Nowadays, many different chemical and herbal agents are available for quick control of bleeding.

**Objectives:**

In this study, we compare the effectiveness of two different types of chemical agents for control of bleeding in an animal model.

**Materials and Methods:**

This research was done comparing two hemostaticagents- ”Chitohem” and “Quikclot”. Ten healthy IR Iranian sheep were chosen and were blindly divided into two different groups. In each of the groups, one of the aforementioned agents was to be applied. First, four main limb arteries of the sheep were dissected linearly and after measuring the volume of bleeding in the first 60 seconds, the chemical agent was applied to the site of bleeding. After that, the duration of bleeding, the volume of bleeding and the secondary blood pressure were measured and compared.

**Results:**

There were no significant differences between the primary features of the animals in two groups (Weight, Baseline Systolic Blood Pressure and Pre-treatment Blood loss). In dependent quantities such as the volume of bleeding after the usage of chemical agents, secondary systolic blood pressure, the results were in favor of “Quick Clot” (*P* < 0.001 for volume of bleeding, *P* = 0.008 for secondary blood pressures and *P* < 0.001 for the necessary time for the bleeding to stop).

**Conclusions:**

In this study, it seems that activity of “Quikclot” in cessation of bleeding of large arterial vessels was slightly better than “Chitohem”. Due to limitations which we had in this study, further studies are necessary to show the actual differences between these agents and their side effects.

## 1. Background

Maintenance of blood circulation is one of the most important goals in primary care of a trauma patient. Uncontrolled bleeding also can lead to multiple problems such hypothermia, infection, multi-organ failure and coagulopathy (1-6).

In the military, management of trauma is more difficult ; there are usually multiple wounds which bleed severely and the environment in which the patients are located is dangerous and devoid of necessary medical supplies (7-9). The majority of bleeding occurs before hospitalization of the patient. Thus, pre-hospital bleeding control is a vital . Several physical methods are used for bleeding control such as using a tourniquet or an air bag, bleeding control with cold or amputation (10). Beside these traditional methods, many chemical agents have been used for bleeding control. The first chemical agents which were used to control bleeding were gelatin, collagen, oxidized cellulose, and bone wax. In one study it was shown that horse collagen has positive effects in bleeding control in patients undergoing simple elective operations(11). These agents also showed various side effects in many previous studies (12). The US Army Institute for Surgical Research described the ideal qualities of hemostatic agents for pre-hospital and combat use (13) These characteristic features are: 1- Capability to stop large vessel arterial and venous bleeding within 2 minutes of application. 2- No requirement for mixing or pre-application preparation. 3- Simplicity of application by the wounded victim. 4- Light weight and durable. 5- Long half-life in extreme environments (cold or rainy weather) 6- Safe to use with no risk of injury or infection. 7- Low cost.

Many studies compare the effectiveness of these new chemical agents. In one animal-model study, effectiveness of a new hemostatic agent with a mineral base has been compared to another agent with a Kaolin-based granular substance (Quikclot) (14). The food and drug administration of USA has recently approved its use. We compare a hemostatic a powder with “Quikclot” in an animal model.

## 2. Objectives

In this study, we compare the effectiveness of two different types of chemical agents for control of bleeding in an animal model.

## 3. Materials and Methods 

This experimental study and was performed from July 2009 to August 2010. In our study 10 full-grown IR Iranian-race sheep ranging in weight between 30 to 40 Kg were used. The health of each animal was checked by a veterinarian before their entrance to the animal laboratory. The factors that were checked for this purpose were appropriate weight, body shape, respiratory rate, heart rate, temperature, and the shape of their teeth. The animals were fed a standard diet for one day and then fasted 18 hours prior to study. On the day of operation, the animals were flanked on a table and the wool of the neck and extremities was shaved. Then, the mentioned sites were disinfected and an angio-catheter (Size 16) was inserted into the left jugular vein. The animal was placed on the operation table in the supine position. After that, the systolic arterial blood pressure of the animal’s limb was indirectly and non-invasively measured and the animal was given Na-Thiopental (15 mg/kg) intravenously for general anesthesia which was followed by maintenance of 2% halothane in oxygen after intubation and mechanical ventilation. At first, the right brachial artery was exposed via a large surgical incision (7-10 cm) made in the upper part of the extremity .The end of the exposed artery was ligated and approximately 3 cm of artery was linearly dissected. In this step, we let the arterial blood flow freely for 60 seconds and then the blood was suctioned and measured.

Then, one kind of powder (Quikclot or Chitohem) was administered at the bleeding site. The secondary blood loss (post-treatment) and the duration of bleeding were measured after the administration of the powder. During these procedures, 5 cc Lidocaine 2% was poured around the artery to prevent the arterial contraction. After the cessation of bleeding, the vital signs of the animals were stabilized by IV-fluid hydration and then similar incisions were done on the other extremity. After performing this for all 4 extremities the cutaneous incisions were closed using silk sutures. Two hours after the first incision, the systolic blood pressures of the animal were again measured. After extubation 600,000 IU Penicillin G was injected for each animal. The animals were kept in the animal lab for 7 days after the operation. The data were analyzed by Mann-Whitney test using SPSS version 14 software.

## 4. Results

In group A “Quik clot” was administered as hemostatic agent and in group B “Chitohem” was used. No significant difference was found in base-line parameters among these two groups (Table 1). Fluctuations were seen in the function of “Chitohem”. In 5/20 incisions Chitohem could not stop the bleeding. Better results for “Quikclot” in comparison to “Chitohem” were prevalent (Table 2). The mean secondary (post-treatment) systolic blood pressure was 65.8 mmHg +/- 2.28 in group A and 58 +/- 1 in group B which show statistical significance (*P* = 0.008). The mean of secondary blood loss was 51.1 cc +/- 4.48 in group A and 63.3 +/- 12.04 in group B which was statistically significant (*P* < 0.001). The duration of the bleeding after the administration of the powder was in group A, 74.5 sec +/- 2.39 and in group B was 86.5 sec +/- 2.6 which was also significant (*P* < 0.001).

**Table 1.** Baseline data

	Group A (Quikclot)	Group B (Chitohem)	Significance
Weight	33.55+/-2.12	33.95+/-2.90	No Significance
Baseline Systolic Blood Pressure	72.4+/- 2.7	73.2 +/- 3.27	No Significance
Pre-treatment Blood loss	91.6+/- 6.16	92.2+/-5.61	No Significance

**Table 2.** Post-treatment Data

	Group A (Quikclot)	Group B (Chitohem)	Significance
Post-treatment Blood loss	51.1+/-4.48	63.3+/-12.04	Significant
Duration of post treatment bleeding	74.5+/-2.39	86.5+/-2.60	Significant
Post-treatment Systolic blood pressure	65.8+/-2.28	58+/-1.00	Significant

## 5. Discussion

In the current study, due to restricted financial resources and also considering ethical issues no placebo agent was used. Instead, we have chosen “Quikclot” powder which is one of the most famous bleeding-control agents to compare with the IR Iran-made powder (Chitohem) (15-17).

According to previous studies, the efficacy of “Quikclot” has been approved for use in combat and emergency settings. This agent has been used in the Iraq war and approved by the Food and Drug administration (FDA) of USA (17). Chitohem could not stop the bleeding in two of the five animal models (5 incisions among 20 in group B). After failure of Chitohem to control the bleeding and after exceeding the secondary bleeding volume by more than 100cc during the procedure, we decided to suture the vessel in order to continue our study on other limbs. .During the procedure we have detected that in the cases of Quikclot administration, higher amounts of thermal reaction in comparison with Chitohem occurred (as a side effect) and it may cause more soft tissue damage and produce problems for patients. This side effect should be checked precisely in future research as such problems can limit to some extent, the use of Quikclot to massive hemorrhage.
